# Onchocerciasis‐associated epilepsy: From recent epidemiological and clinical findings to policy implications

**DOI:** 10.1002/epi4.12054

**Published:** 2017-04-26

**Authors:** Robert Colebunders, Alfred K. Njamnshi, Marieke van Oijen, Deby Mukendi, Jean Marie Kashama, Michel Mandro, Nolbert Gumisiriza, Pierre‐Marie Preux, Patrick Suykerbuyk, Richard Idro

**Affiliations:** ^1^ Global Health Institute University of Antwerp Antwerp Belgium; ^2^ Neurology Department Central Hospital Yaoundé Faculty of Medicine and Biomedical Sciences the University of Yaoundé I Yaoundé I Republic of Cameroon; ^3^ Department of Neurology Academic Medical Center Amsterdam the Netherlands; ^4^ Neuro‐psycho‐pathological Centre University of Kinshasa Kinshasa Democratic Republic of the Congo; ^5^ Provincial division of Health of Ituri Ministery of Health Bunia Democratic Republic of the Congo; ^6^ Busitema University Mbale Uganda; ^7^ Tropical Neuro‐epidemiology unit University of Limoge Limoges France; ^8^ College of Health Sciences Makerere University Kampala Uganda; ^9^ Nuffield Department of Medicine Centre for Tropical Medicine and Global Health University of Oxford Oxford United Kingdom

**Keywords:** Epilepsy, Nodding syndrome, Nakalanga syndrome, Ivermectin, Prevalence, Incidence

## Abstract

A high prevalence of epilepsy is reported in many onchocerciasis‐endemic regions. In this paper we discuss recent epidemiological and clinical aspects as well as public health implications of onchocerciasis‐associated epilepsy (OAE) and propose a strategy to reduce the burden of disease. OAE probably presents in a variety of clinical manifestations, including the nodding syndrome and the Nakalanga syndrome. The most common clinical presentation, however, is generalized (primarily tonic‐clonic) seizures. A characteristic of OAE is the onset of seizures between the ages of 3 and 18 years and clustering in certain families and villages close to rapid‐flowing black‐fly‐infested rivers. A strategy combining active surveillance for epilepsy with early treatment with antiepileptic drugs and prevention of onchocerciasis by increasing the geographical and therapeutic coverage of community‐directed treatment with ivermectin (CDTi) may considerably decrease the burden of disease.

**Key Points**

- Onchocerciasis‐associated epilepsy (OAE) occurs clustered in certain families and villages close to rapid‐flowing black‐fly‐infested rivers
- OAE seizures generally start between the ages of 3 and 18 years in previously healthy children
- Clinical presentations of OAE include the nodding syndrome and the Nakalanga syndrome
- Active surveillance for epilepsy in onchocerciasis‐endemic regions with early antiepileptic treatment and increasing the coverage of community‐directed treatment with ivermectin may considerably decrease the burden of disease

An estimated 70 million people in the world are affected by epilepsy, with about 2.4 million people diagnosed each year.^1^, ^2^ Epilepsy prevalence varies largely among continents and countries, with a considerably higher prevalence in populations in low‐ and middle‐income countries.^2^, ^3^ Birth trauma, traumatic brain injury, cerebral vascular disease, brain tumors and bacterial brain infections are well‐known causes, but parasitic infections, such as cerebral malaria, neurocysticercosis, echinococcosis, and onchocerciasis, are also known to be associated with epilepsy.^3^ In this paper we discuss epidemiological and clinical aspects as well as public health implications of onchocerciasis‐associated epilepsy (OAE) and propose a strategy to reduce the burden of disease.

Onchocerciasis, also known as river blindness, is a parasitic disease caused by the filarial worm *Onchocerca volvulus* (Ov) transmitted by black flies of the genus *Simuliidae*. Today it is estimated that 37 million people are infected by Ov, of whom 99% live in Africa.^4^ In infected persons, the adult female worms form subcutaneous nodules and release thousands of microfilariae daily, leading to itching, dermatitis, blindness (all well‐known complications of onchocerciasis),^5^ and epilepsy.^6^

## Epidemiological Aspects

The link between epilepsy and onchocerciasis was first reported in 1938 by the Mexican physician Casis Sacre, who described a syndrome characterized by epileptic seizures, stunted growth, and mental retardation in patients with onchocerciasis in Chiapas and Oaxaca, Mexico.^7^ In Africa, the association between epilepsy and onchocerciasis was first documented in a population‐based epidemiological study by Boussinesq et al.^8^ in the Mbam valley in Cameroon in 1991–1992. In this study, the prevalence of epilepsy increased with decreasing distance to the Mbam River, a breeding site for black flies, and with increasing community microfilariae load.^8^ Since then, similar associations have been documented in studies from many other African countries.^9^, ^10^, ^11^, ^12^ In particular, a huge case‐control study conducted in several countries of sub‐Saharan Africa found an increased prevalence of epilepsy with onchocerciasis, with an odds ratio of 2.2 (confidence interval [CI] 95%: 1.6–3.2).^3^ A meta‐analysis of African population‐based surveys showed a variation in epilepsy prevalence consistent with onchocerciasis prevalence, with epilepsy prevalence being increased, on average, by 0.4% for each 10% increase in onchocerciasis prevalence.^13^ There have also been case‐control studies that did not show the association between onchocerciasis and epilepsy,^14^ but these studies were performed in areas of low onchocerciasis endemicity^13^ or were not able to show an association because cases and controls were matched for ivermectin exposure.^15^ Of note is that none of the case‐control studies was carried out on incident cases. In all of these studies, cases developed epilepsy many years earlier, and this makes it difficult to identify risk factors preceding the development of epilepsy. Acute symptomatic seizures were not considered as epilepsy in the studies in which the authors of this paper were involved.

In the 1960s a distinctive epilepsy syndrome characterized by head nodding, the nodding syndrome (NS),was first described in an onchocerciasis‐endemic region in Tanzania (in children from a few villages in the Mahenge Mountains) by Jilek‐Aall.^16^ Other characteristics of NS include stunted growth, as in the Nakalanga syndrome,^17^, ^18^ and cognitive decline. Since then, NS‐ and Nakalanga‐like clinical features have been reported in other onchocerciasis‐endemic areas in Liberia,^19^ West Uganda,^20^ Burundi,^21^ and possibly the Central African Republic, Ethiopia, Mali,^22^ and Cameroon.^13^ NS epidemics have been observed in onchocerciasis‐endemic regions in South Sudan (onset around 1990)^23^ and in northern Uganda (onset around 2002).^24^ Case‐control studies in the two countries demonstrated a statistically significant higher prevalence of onchocerciasis in individuals with NS than in controls.^23^, ^24^

Although the association among epilepsy, NS, and Ov infestation seems apparent, the pathophysiological mechanism is not clear. In recent studies Ov DNA was never isolated from cerebrospinal fluid (CSF) in patients with NS/OAE.^23^, ^24^, ^25^, ^26^ However, several of the patients enrolled in these studies had taken ivermectin (the antiparasitic drug commonly used to treat onchocerciasis) in the past. In earlier studies, before the use of ivermectin, several investigators had reported the presence of microfilariae in the CSF of patients with onchocerciasis: Hisette in 1932 in Congolese patients with ocular onchocerciasis^27^ and Casis Sacre in 1938 in Mexican patients. In 1959 dead and live microfilariae were found by Mazotti in CSF of patients with onchocerciasis treated with diethylcarbamazepine.^28^ In 1976 Duke et al.^29^ also observed microfilariae in the CSF of heavily infested patients.

Although these findings would suggest a direct effect of microfilaria, another explanation for the association could be the occurrence of an autoinflammatory response induced by antibodies to Ov cross‐reacting with neuron proteins.^30^ This recent paper seems to support our earlier pilot study that observed antibodies to voltage‐gated potassium channels in neurons.^31^

**Figure 1:**
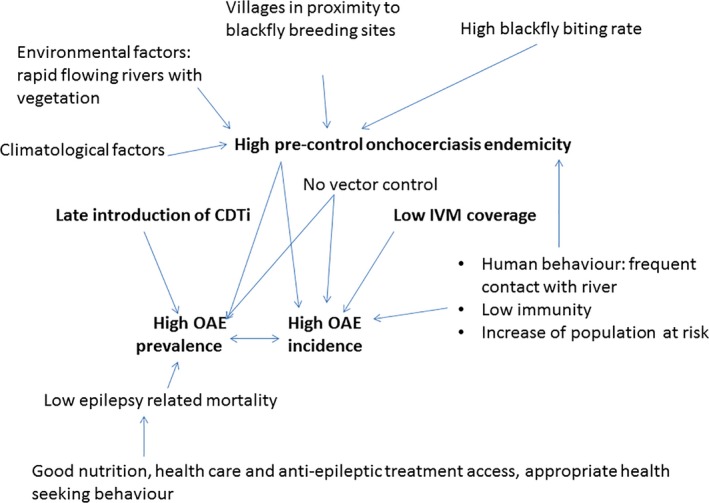
Many factors influence the prevalence and incidence of onchocerciasis‐associated epilepsy.

Because Ov may directly or indirectly cause epilepsy, the prevalence and incidence of epilepsy are determined by all the factors that influence the level of the preonchocerciasis control endemicity (Fig. 1).

Onchocerciasis endemicity is influenced by environmental factors: the presence of fast‐flowing rivers with rapids and vegetation at their border; climatological factors: a more constant weekly rate of rainfall may increase the risk for onchocerciasis transmission; the proximity of the village to black fly breeding sites and black fly biting rates: human biting rates in a village may decrease if there is another village closer to the river or if there is a lot of cattle in the village;^32^ human behavior: frequent river contact particularly during the daytime when black flies are most active; size of the population at risk: the establishment in northern Uganda of very large internally displaced person camps close to black‐fly‐infested rivers and population growth in villages close to blackfly breeding sites such as in Mvolo in South Sudan may have played a role in causing NS epidemics;^33^ and degree of immunity among the population: a low cattle:human ratio may lead to increased Ov infestation. Indeed, infection with *Onchocerca ochengi*, a species prevalent in cattle but transmissible by black flies to humans, may not cause the disease but may cause the creation of antibodies that provide some protective immunity against Ov infestation;^34^ malnutrition and untreated coinfections during episodes of war such as in northern Uganda and South Sudan may have rendered children more vulnerable to heavy infestation with Ov. Exposure to multiple parasites also may increase the prevalence of epilepsy.^35^

Today, onchocerciasis endemicity mainly depends on the quality of the onchocerciasis control program in the area. Between 1995 and 2015, the African Program for Onchocerciasis Control (APOC) coordinated the implementation of community‐directed treatment with ivermectin (CDTi) programs in onchocerciasis‐endemic areas of 22 African countries.^36^ Activities toward the control of onchocerciasis keep expanding in the frame of the World Health Organization (WHO) Expanded Special Project for Elimination of Neglected Tropical Diseases (ESPEN) that was launched in May 2016.^37^ By controlling the blackfly by larviciding its breeding sites in fast‐flowing rivers, a remarkable reduction of onchocerciasis transmission has been achieved in the past 20 years. However, despite the success and the effectiveness of these targeted intervention programs, certain zones still remain unreached by CDTi, because of local insecurity due to armed conflicts in the endemic region.

It is known that ivermectin rapidly reduces the body's microfilariae load, thus eliminating the potential trigger that is associated with epilepsy. It therefore appears plausible that high ivermectin therapeutic coverage will decrease the incidence of OAE fairly rapidly. The NS epidemic in northern Uganda started to decrease after 2008 when a limited number of people started ivermectin treatment. In a case‐control study in 2009 in the Kitgum‐Pader Districts 33% of NS cases and 24% of controls had taken ivermectin.^24^ In 2012 the NS epidemic stopped (no new NS cases appeared) within a year after implementing biannual CDTi and larviciding rivers.^33^ In 2014, in another case‐control study in the Kitgum District 76% of NS cases and 77% of controls had taken ivermectin.^38^ In the Democratic Republic of the Congo (DRC), a first small case‐control study suggested that ivermectin intake may protect against the development of epilepsy in onchocerciasis‐endemic areas,^25^ and in a more recent study of 96 cases and 96 controls, matched for village, age, and sex, this finding was confirmed (R. Colebunders, personal communication).

Late introduction of CDTi in onchocerciasis hyperendemic areas, as was the case in certain districts in northern Uganda, has led to high prevalence of OAE. OAE prevalence is also influenced by mortality. Where there is access to adequate health care and antiepileptic treatment, epilepsy‐related mortality will be low and therefore the prevalence of epilepsy may only decrease slowly despite an effective CDTi program. In this situation, because children with OAE will become adults and very few new children will develop OAE, the highest prevalence of epilepsy will be observed among the age group between 20 and 30 years and not among the 10‐ to 20‐year age group before the introduction of CDTi.^33^ In most places in Africa, epilepsy is associated with increased mortality^39^ because children with epilepsy often die at a young age from drowning, burn injuries, status epilepticus (itself due to lack of access to anti‐epileptic treatment), or neglect, malnourishment, or infections.^40^

## Clinical Aspects

OAE manifests with a variety of seizure types and degrees of severity.^41^ In Tanzania, Uganda, and South Sudan, a possibly distinctive form of OAE has been described as NS.^42^ NS is a debilitating epileptic disorder developing in children 3–18 years old.^42^ NS seizures are characterized by a brief loss of muscle tone in the neck (atonic seizure), leading to repeated head nodding, which gave the disease its name.^43^ Cognitive decline and stunted growth in formerly normally developing children are other characteristics of the disease.^23^, ^43^, ^44^ Repeated seizures are probably the main cause of cognitive decline in persons with OAE. In northern Uganda, since the start of antiepileptic treatment and nutritional and psychosocial support, it has been shown that NS may not be an invariably progressive disease. Several children were able to return to school. This suggests that if timely and adequate treatment and care are provided, cognitive decline can be prevented.

Persons with OAE often present with onchocerciasis‐related dermatological manifestations but rarely with blindness. To become blind, most likely a much longer exposure to the Ov infection is required.^45^

**Figure 2:**
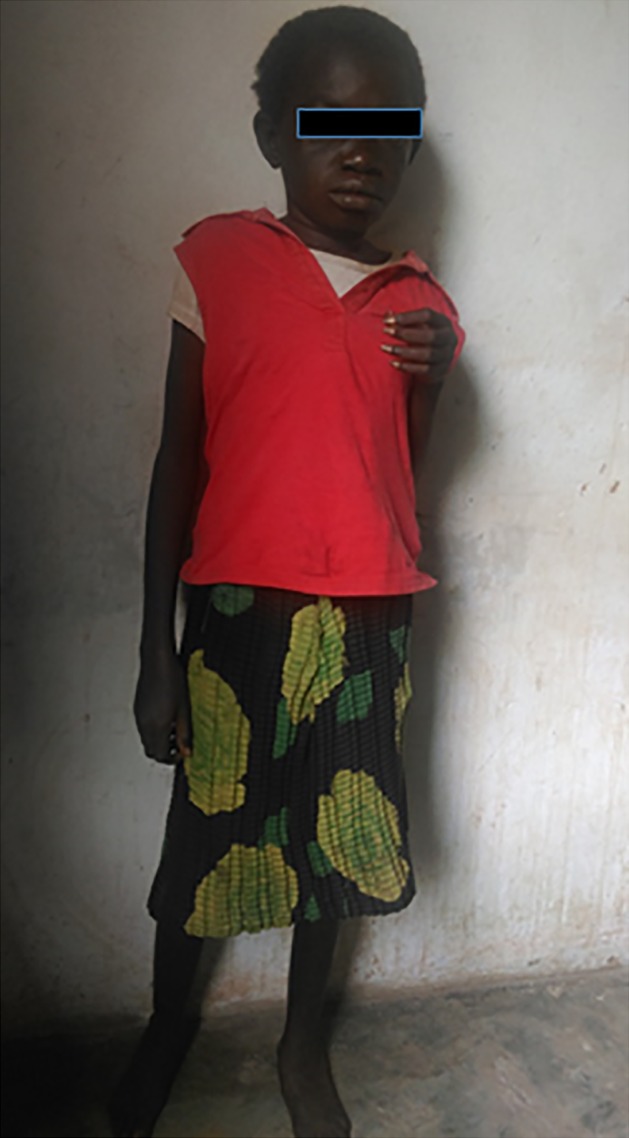
Woman, with Nakalanga syndrome, 26 years old, from an onchocerciasis‐endemic region in the Democratic Republic of the Congo.

The Nakalanga syndrome is another clinical condition observed in onchocerciasis‐endemic regions that is associated with epilepsy. The Nakalanga syndrome was first described in 1966 among a population who migrated to the Mabira Forest, Buikwe District, in the central region of Uganda, which was at that time an onchocerciasis‐endemic region.^46^, ^47^ The Nakalanga syndrome is characterized by severe stunting and absence or delayed development of external signs of sexual development.^17^, ^46^ Nakalanga features have been described in several other onchocerciasis‐endemic regions.^21^, ^48^, ^49^ During epilepsy prevalence surveys performed between 2014 and 2016, in the DRC several persons with stunted growth, absence of external signs of sexual development, cognitive impairment, and epilepsy were observed in onchocerciasis‐endemic areas in the Bas Uélé, Tshopo, and Ituri Provinces. For example, a pronounced form of Nakalanga was observed in a of 26‐year‐old women in Tshopo Province. She had the appearance of a child (weight 26 kg, height 1.27 m), without any external signs of sexual development. She had developed tonic‐clonic seizures at the age of 16, was cognitively impaired, and had only reached third grade of primary school. A Skin snips taken from the left and right italic crests showed the presence of microfilariae (parasite densities of 10 and 33 microfilariae/mg skin, respectively; Fig. 2).

Most, but not all reported cases of Nakalanga had a history of epilepsy. It is, however, not clear whether there was no history of epilepsy or whether it was only not reported by the authors. All Nakalanga cases observed by us (RC, RI, MM, and DM) had a history of epilepsy. Head nodding was not reported in the limited number of persons with Nakalanga described in the literature, but in most cases, the type of epilepsy was not specified.^18^ Both NS and the Nakalanga syndrome seem to disappear from the area when an onchocerciasis control program is implemented.^50^

Most patients with OAE present with generalized primarily tonic‐clonic seizures, clinically not different from seizures of another etiology. The main differences between OAE and other forms of epilepsy are the time of onset, between the ages of 3 and 18 years (mean age 8–12 years), the fact that persons with epilepsy live in villages with high Ov transmission, and the fact siblings in the family often are also affected by epilepsy.^40^ The reason why some children present with generalized tonic‐clonic seizures without other symptoms and others present with NS or features of Nakalanga syndrome is not clear. In northern Uganda and in South Sudan, we observed clustering of these three different clinical presentations in the same families and in the same villages, suggesting that they are indeed triggered by a common factor. This trigger seems to be an Ov infestation. The degree of Ov infestation, the time the children were exposed to Ov, whether seizures are adequately treated with antiepileptic drugs, and certain cofactors, such as malnutrition, may explain the difference in clinical presentation.^50^ Children with Nakalanga syndrome are known to be heavily infested with Ov, and the first signs that a child will develop the Nakalanga syndrome are already observed in the second or third year of life.^47^ These children were probably infected with Ov at an early age, when their brains were still developing. It is possible that other children, slightly less exposed to Ov and/or exposed later in life, may develop NS (the mean age for developing NS in children in Tumango, in northern Uganda was 7.6 years^24^). Children less exposed to Ov and much later in life may develop epilepsy with minimal or no cognitive impairment and no decrement of growth or sexual development (the mean age of developing tonic‐clonic seizures in the DRC was 11 years^9^).

## Case Definitions

In 2012 a case definition for NS was proposed during a meeting coordinated by the WHO and Ugandan Ministry of Health in Kampala.^51^ This case definition was found to be complicated for use in epidemiological surveys.^52^ Moreover, there is no case definition for other forms of epilepsy associated with onchocerciasis, including the Nakalanga syndrome. Today, because of the increasing evidence of an association between NS and onchocerciasis, the 2012 WHO case definition of NS may need to be updated. Recently, a simple point‐of‐care test, the Ov16 rapid antibody test, became available.^50^, ^53^ Including a positive Ov16 rapid antibody test in the case definitions of onchocerciasis‐associated conditions could be considered.

Proposed clinical case definitions for OAE, NS, and Nakalanga syndrome are as follows: **OAE** (need to meet the following five criteria)*

1. Person with epilepsy living in an onchocerciasis‐endemic region
2. Onset of epilepsy between the ages of 3 and 18 years
3. Geographical clustering of persons with epilepsy in the village, or brother or sister with epilepsy
4. No obvious cause for the epilepsy**
5. Normal neurological development before the onset of epilepsy

**NS** = OAE + nodding of the head with episodes of decreased responsiveness

**Nakalanga syndrome** = OAE + important growth retardation and delay or no development of external signs of sexual development without an obvious cause for the growth retardation. If no history of epilepsy, one of the following additional criteria is needed: Ov16 seropositivity, or skin test positivity (presence of microfilariae or Ov PCR positive), or presence of onchocerciasis clinical manifestations (characteristic skin lesions and/or nodules).

*In case the person has never taken ivermectin, one of the following additional criteria is needed: Ov16 seropositivity, or skin test positivity (presence of microfilariae or Ov PCR positive), or presence of onchocerciasis clinical manifestations (characteristic skin lesions and/or nodules).

**To exclude other causes of epilepsy may be difficult in most onchocerciasis‐endemic regions because complementary exams may not be feasible. Therefore, without such exams a person meeting the five criteria should be considered as a probable OAE case.

## Public Health Importance

The exact burden of disease attributed to OAE is currently unknown but seems to be considerably high because of the number of people at risk. Indeed, if we consider that the onchocerciasis infection is poorly controlled in 30% of the 37 million people infected,^4^ and that 1% of those develop epilepsy (equivalent to the approximate excess prevalence of epilepsy over nononchocerciasis areas), the number of OAE cases could be more than 100,000.^50^ In Mvolo, a village in South Sudan, one in six children suffer from epilepsy and at least 50% of the families have at least one child with epilepsy.^40^ Untreated OAE may lead to further cognitive and physical decline due to uncontrolled seizures and possibly neglect.^44^ The psycho‐socio‐economic importance of OAE in severely affected communities is enormous.^54^ Many OAE‐affected children have psychiatric problems. Girls with OAE with an intellectual disability are at risk of being sexually abused. Children often die at a young age because they drown in a river or fall in a fire and sustain severe burns. Children with OAE may also be affected by onchocerciasis skin disease that causes intense itching that prevents them from sleeping.^54^ Some of the affected children require constant care because they may wander off and get lost.^54^ This interferes with the guardians’ day‐to‐day socio‐economic activities reducing the families’ income. When they become children with untreated OAE may not be able to contribute to the family income or take care of older family memebers.

OAE only occurs in remote rural regions of Africa, where people with epilepsy often have no continuous access to antiepileptic treatment or to basic care for epilepsy‐associated complications such as burn wounds. Local health care workers are often not sufficiently trained to treat persons with epilepsy, and there are virtually no neurologists. Moreover, children with epilepsy risk being deprived of education. In many cultures in Africa, there is a belief that persons with epilepsy are possessed by evil spirits and the entire family may suffer from social isolation through stigmatization.^55^

By reducing the disease burden of OAE, the socio‐economic status of affected families in Africa will be positively affected, which in turn will ultimately benefit society as a whole. In certain communities in onchocerciasis‐endemic regions, ivermectin treatment coverage remains suboptimal. If we can prove that ivermectin can reduce the incidence of epilepsy in onchocerciasis‐endemic regions this will increase the willingness of populations to carefully take the ivermectin every year and will motivate public health officials and funders to strengthen CDTi programs. Ivermectin once a year may not be enough to protect a population against OAE because microfilariae may reappear several months after the intake of the drug. In addition, options of providing treatments of a shorter duration may also be explored. This may ultimately lead to the elimination of onchocerciasis^33^ and potentially OAE.

## Policy Implications

**Figure 3:**
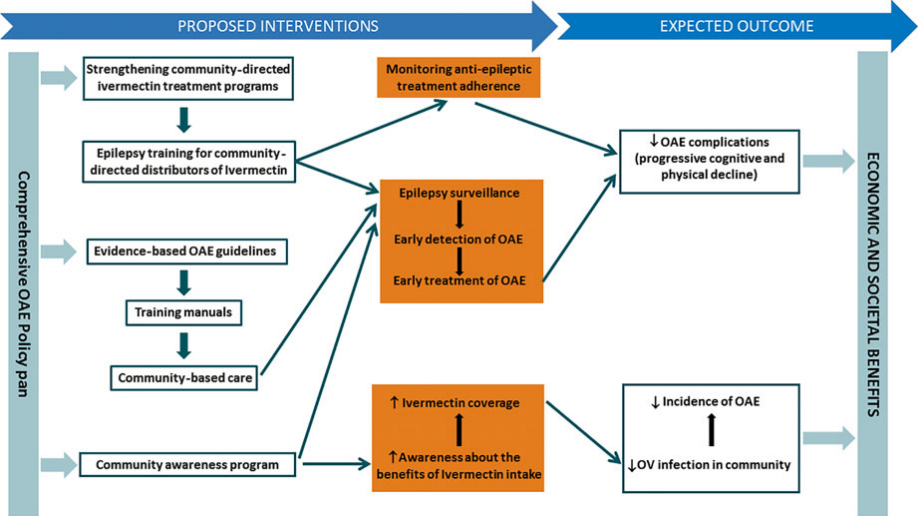
Proposed interventions to decrease the impact of onchocerciasis‐associated epilepsy.

Immediate action is needed because OAE is catastrophic for entire villages in many remote onchocerciasis‐endemic regions.^40^ OAE policy plans are required and should include the following activities. Of course, these could also be of benefit for other types of epilepsies (Fig. 3).

1. An epilepsy surveillance system using trained community health workers. Such a system is important for public health officials not only for planning interventions and resource needs but also for improving patient care. It could be set up by using the CDTi distributors.
2. When a person with seizures is found in a village, the local CDTi distributor needs to inform and report by text message to a local health care worker who has been trained to diagnose and treat epilepsy. First, an acute cause of seizures (such as encephalitis/meningitis, metabolic disturbances or intoxications) should be excluded, according to the Mental Health GAP guidelines.^56^ If there is no reason to suspect an acute cause and the diagnosis of epilepsy is confirmed, common underlying causes such as birth asphyxia and trauma, head injury, history of infection of the brain, and family history of seizures should be explored by taking the patient's history and doing a complete neurological examination. In remote onchocerciasis‐endemic regions, persons often present with seizures of many years without any evidence for a concomitant acute or progressive chronic disease or for a focal neurological deficit. Such an individual could be treated with antiepileptic drugs (AEDs) without performing additional tests. Whether a positive Ov16 antibody test could be useful in the work‐up of epilepsy in onchocerciasis‐endemic regions to decide whether a person should be transferred to a specialized center to exclude other causes of the epilepsy needs to be investigated.
3. Patients with OAE should receive uninterrupted treatment with good‐quality AEDs, and treatment adherence should be monitored. First, the setup of such a system requires the decentralization of epilepsy services. Second, uninterrupted access to AEDs at lower‐level health units is needed using a public health approach with simplified low‐cost and child‐ appropriate antiepileptic treatment regimens. Last, this process requires a task shift: the care of patients with OAE, after diagnosis, could be managed by primary health care workers assisted if necessary by medical doctors or neurologists.^57^ Training manuals are needed for these health care workers, school teachers need to be taught how to work with children with OAE, and a program to prevent and treat burns in children with OAE needs to be elaborated.
4. There is an obvious need for a program to prevent OAE by strengthening CDTi programs. Health zones with low ivermectin coverage need to be identified and reasons for low coverage need to be investigated. Some individuals are not taking ivermectin because they are not well informed about the drug's benefits and because they are afraid of the side effects. Therefore, a community program to raise awareness and address misconceptions is needed to increase the coverage of ivermectin and to fight epilepsy‐associated stigma and discrimination. This could be done through health education and a community mobilization program involving persons who suffered from epilepsy in the past but who now are living normal lives thanks to AEDs and onchocerciasis treatment.

Challenges to obtaining optimal coverage by ivermectin include poverty, ignorance, insecurity, and war. Most of the successes with mass drug administration to eliminate onchocerciasis have been observed in countries and communities with higher living standards, such as in South America.^58^ Therefore, the eradication of onchocerciasis will require efforts beyond an entirely pharmacologic intervention to include strengthening of the health care system and a comprehensive socioeconomic and political approach.

## Conclusion

OAE is a neglected disease that occurrs in remote areas in Africa and that affects very poor populations. The incidence of OAE could be significantly reduced by strengthening onchocerciasis control programs. The suffering of individuals with OAE and affected families could be reduced by timely antiepileptic treatment. To implement such a policy, partnerships among scientists, affected communities, advocacy groups, health care workers, Ministries of Health, WHO, nongovernmental organizations, the pharmaceutical industry, the tech industry and funding organizations are needed.

## Disclosure

None of the authors has any conflict of interest to disclose. We confirm that we have read the Journal's position on issues involved in ethical publication and affirm that this report is consistent with those guidelines.
